# Genetic Diversity and Differentiation of *Dendrocalamus membranaceus* (Poaceae: Bambusoideae), a Declining Bamboo Species in Yunnan, China, as Based on Inter-Simple Sequence Repeat (ISSR) Analysis

**DOI:** 10.3390/ijms13044446

**Published:** 2012-04-10

**Authors:** Han-Qi Yang, Man-Yun An, Zhi-Jia Gu, Bo Tian

**Affiliations:** 1 Research Institute of Resource Insects, Chinese Academy of Forestry, Kunming 650224, China; E-Mails: yanghanqikm@yahoo.com.cn (H.-Q.Y.), anmanyun@yahoo.com.cn (M.-Y.A.), guzhijia139@126.com (Z.-J.G.); 2 Laboratory of Tropical Plant Resource Science, Xishuangbanna Tropical Botanical Garden, Chinese Academy of Sciences, 88 Xuefu Road, Kunming 650223, China

**Keywords:** *Dendrocalamus membranaceus*, genetic variation, ISSR, population structure

## Abstract

*Dendrocalamus membranaceus* Munro is a woody bamboo with a high economic and ecological value that often occurs as natural stands, such as in the large-scale forested areas of China’s Yunnan Province. Due to its overexploitation, the habitat of *D. membranaceus* in Yunnan has been dramatically reduced, and the quality of the stands has declined. As a preliminary analysis in considering the effective protection for these germplasm resources, we assessed the genetic diversity of 12 natural populations in Yunnan, using inter-simple sequence repeat (ISSR) markers. From 10 ISSR primers, we generated 155 bands, of which 153 were polymorphic (98.71%). Compared with other species in the genus, this species demonstrated a greater genetic diversity (*S* = 0.349) and lower genetic differentiation (*G*_ST_ = 0.252). Our analysis of molecular variance revealed that the genetic differentiation among the populations is significant. A large proportion of the genetic variation (78.95%) resides among the individuals within populations, whereas only 21.05% are found among populations. Mantel tests indicated no significant correlation between genetic and geographic distances among the populations. Given the low sexual reproducibility and characteristics of monocarpic plants, we recommend implementing *in situ* conservation measures for all of the *D. membranaceus* populations in Yunnan and collecting sufficient samples for *ex situ* conservation. Furthermore, the conservation area should be extended to its main natural habitats, the Lancang-Mekong River Valley.

## 1. Introduction

*Dendrocalamus membranaceus* Munro is one of the most frequently occurring, clump-forming woody bamboos (with pachymorph rhizomes) in Southeast Asia. It is naturally distributed in Laos, Myanmar, Northern Vietnam and Northern Thailand [1–3] in addition to China’s Yunnan Province, especially along the Lancang-Mekong River Valley [4]. This species can grow to 20 m tall and 10 cm in diameter, and is economically important as a vegetable crop and as raw material for furniture, construction, and industrial paper pulp. Its natural habitat is a tropical mixed deciduous or monsoon forest below 1000 m elevation [3,4].

In 1995, Yunnan Province had approx. 70,000 hm^2^ of natural *D. membranaceus* forest, which provided important support for the local ecosystem, including uses as a food resource (bamboo shoots and young culms) and as habitat for the wild Asian elephant [5]. As a priority species, this bamboo has been crucial in efforts toward the protection of local soils and biodiversity in the southern and western portions of the province [6]; however, because most of its native habitat does not exist in nature reserves, this species has long been overexploited in Yunnan. For example, during the past two decades, the development of tropical agriculture plantations for rubber [7] and tropical fruits has meant that the area traditionally comprising stands of *D. membranaceus* has been dramatically reduced, to less than 30,000 hm^2^ in 2008, with obvious degradation of the quality of the remaining forest [8]. This has raised great environmental questions about water loss, soil erosion, and a decline in biodiversity [6,7]. Therefore, protective measures, in addition to germplasm collections, are desperately needed.

Woody bamboos are presumably ancient polyploids, and *Dendrosalamus* is regarded as hexaploid [9]. According to the Scientific Database of China Plant Species [10], the chromosome number of *D. membranaceus* collected from Yunnan is 2*n* = 70. However, the chromosome number of paleotropical woody bamboo is generally variable, and 2*n* = 70 ± 2 is the most frequently found in *Dendrocalamus* [11]. If x = 12, *D. membranaceus* is most likely hexaploid. Recent work using dominant genetic markers has shown that plant polyploids possess a high genetic diversity [12,13]. However, *D. membranaceus* has been subjected to a severe reduction in its natural distribution, and, in general, woody bamboos reproduce clonally, two conditions that can decrease the levels of genetic variation. Thus, *D. membranaceus* is a good model organism for understanding how bamboos genetically change under declining situations.

Although a strict reliance on vegetative features for the traditional identification of bamboo species and cultivars has seriously limited the ability of researchers to examine their genetic variation and differentiation [14–17], molecular-marker approaches are now being adopted, including random amplified polymorphic DNA (RAPD) [18], simple sequence repeats (SSRs) [19], and inter-simple sequence repeats (ISSRs) [20]. Because the ISSR technique is easily applied, identifies high polymorphism and displays acceptable reproducibility, it is widely used in current studies of population genetics [21–23]. In this study, we employed ISSRs to estimate the degree of genetic variation and differentiation among 12 populations of *D. membranaceus* in Yunnan Province, China (Table 1). Our objective was to use this information to help understand the genetic background of *D. membranaceus* and also to provide a reference for the incorporation of genetic resources when devising programs for species protection and breeding.

## 2. Results and Discussion

### 2.1. Genetic Diversity within Populations of *Dendrocalamus membranaceus*

**Figure 1:**
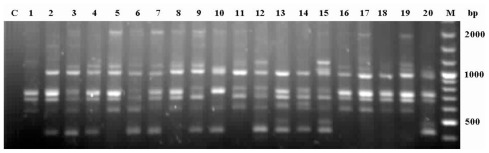
Genetic profile of Mengyang (MY) population using primer UBC810 (as described in Table 2). Lane C represents the blank control and lanes 1–20 represent template DNA for each individual from MY.

Of the 79 ISSR primers used, 10 produced polymorphism; overall, the band reproducibility of these 10 primers is 93% (Table 2). Figure 1 presents the polymorphic fingerprinting pattern for the Mengyang (MY) population, as generated by ISSR primer UBC810. The 10 primers produced 155 reproducible bands in 240 clumps, for an average of 15.5 bands per primer. We considered each clump as a potential genet and the culms within as ramets of a clone, according to McClure [24]. Of the 155 bands, 153 were polymorphic (99%) at the species level. The average percentage of polymorphic bands (PPB) was 48.06%, ranging from 39% (Xingping, XP and Guanlei, GL) to 55% (Gengma, GM) at the population level (Table 3). Assuming a Hardy-Weinberg equilibrium, the expected diversity was estimated to be 0.164 at the population level and 0.219 at the species level. Shannon’s indices of diversity were 0.249 (population) and 0.349 (species). Among our 12 populations, GM exhibited the highest degree of variability (expected heterozygosity, *H*_E_ = 0.186; Shannon’s diversity index, *S* = 0.279), whereas GL had the lowest (*H*_E_ = 0.146, *S* = 0.216; Table 3).

Furthermore, a high level of genetic diversity was found for this species (PPB = 99%, *H*_E_ = 0.219, *S* = 0.349). By comparison, the mean within-population gene diversity among monocotyledons is 0.144 for Nei’s expected heterozygosity [25].

The high genetic diversity detected for *D. membranaceus* may be a consequence of its evolutionary history and the geological development of South Yunnan. *D. membranaceus* is most likely an ancient hexaploid [9,11], and polyploid taxa typically display a great genetic diversity, both in terms of heterozygosity and the number of bands amplified [13]. However, the habitat of the species has not suffered major geological disasters since the Tertiary period. The region is also warm and humid and provides the appropriate conditions for the reproduction of tropical bamboos [26]. Moreover, the natural populations were not, as a whole, disturbed by human activities until recently [5,6]. Therefore, we speculate that (1) the ancestors of *D. membranaceus* have accumulated abundant genetic diversity within an extensive and continuous distribution in Yunnan; (2) the existing population retains the genetic diversity from their ancestors; and (3) the habitat fragmentation caused by human activities does not perturb the genetic base of the existing *D. membranaceus* populations.

### 2.2. Genetic Structure and Differentiation Among Populations

The results from our analysis of molecular variance (AMOVA) showed that a large proportion of the genetic variation (78.95%) existed among individuals within the populations, and that 21.05% resided among the populations. The genetic differentiation among the populations was significant (*p* < 0.001) (Table 4). Consistently, Nei’s [27] estimator of population substructure also indicated a moderate level of population differentiation (coefficient of gene differentiation, *G*_ST_ = 0.252). Furthermore, 24 private bands were found in eight populations (Table 3), implying that some genetic difference existed among the populations.

**Figure 2:**
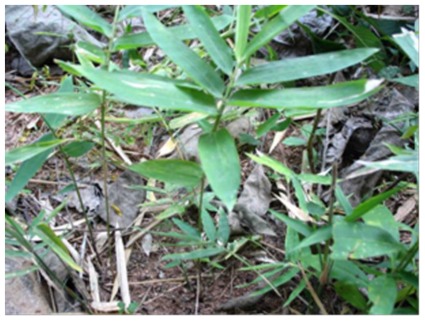
Young seedlings *of Dendrocalamus membranaceus* in Jinghong (JH) population.

Many factors can determine the genetic structure of plant populations, including the reproductive biology [28], and gene flow. Many RAPD- and sequence tagged microsatellite sites (STMS)-based analyses showed that long-lived, out-crossing taxa retained most of their genetic variability within populations [29]. The woody bamboos have a long vegetative phase of 20–150 years [1–3], and are typical long-lived species of the grass family. As one of those critical influences, the out-crossing of a plant species tends to explain 10 to 20% of the genetic variation among populations, whereas the selfing of a species leads, on average, to 50% variation between populations [25]. *D. membranaceus* can reproduce via seed in the wild, although this phenomenon is rare, and the rate of seed set is low [3,16]. We found a few young seedlings in the Jinghong (JH) population (Figure 2). Furthermore, studies on the floral biology have indicated that *D. membranaceus* is likely anemophilous and prone to be an out-crosser [16], which also was supported by the genetic differentiation (*G*_ST_ = 0.252) that was similar to the average of out-crossing species (*G*_ST_ = 0.22) [29].

Therefore, extensive longevity and predominant out-crossing may be two important factors for the genetic structure of the existing *D. membranaceus* in Yunnan.

### 2.3. Genetic Distances and UPGMA Analysis

**Figure 3:**
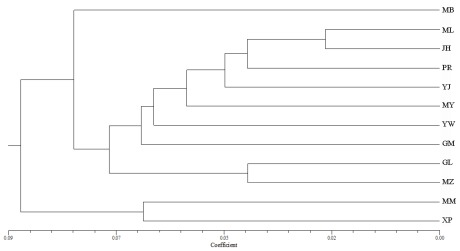
UPGMA dendrogram based on Nei’s (1972) genetic distances among 12 populations of *Dendrocalamus membranaceus*.

**Figure 4:**
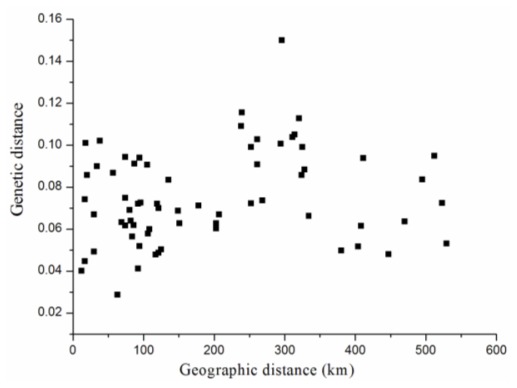
Correlation between geographic distance and genetic distance for 12 populations of *Dendrocalamus membranaceus*.

Our UPGMA tree (Figure 3), based on the values for the genetic distance *D*, revealed that the 12 populations could be separated into two clusters: XP plus Mengman (MM) and the other 10. We found no agreement with the geographic distance, which was supported by our Mantel test results, demonstrating that *D* was not significantly correlated with the geographic distance (*r* = 0.197, *p* = 0.184) (Table 5, Figure 4). This outcome could have reflected the possibility that the examined populations of *D. membranaceus* still represented the abundant genetic variation of their ancestors. In fact, this tendency is reinforced by the naturally long vegetative phase and low seed set over the life cycle, which are typical features of woody bamboos [11,30,31]. Another possibility may be that, as an economic importance species, *D. membranaceus* most likely has undergone human-mediated movement of genotypes among partial or complete distribution areas, especially within those populations comprising small areas, such as XP, GM and Yingjiang (YJ).

### 2.4. Implications for the Development of *D. membranaceus*

The natural resources of *D. membranaceus* in Yunnan Province have been overexploited for many years, leading to the deterioration of its habitat [6,8]. The same situation has also occurred in other regions of the Lancang-Mekong River Valley, such as Myanmar and Northern Vietnam [7]. Although bamboo is of important significance for the local ecological and agricultural systems, the increasing demand for its culms and shoots and the dramatic declines in its native habitats and stand quality indicate that further research that focuses on its germplasm collection and conservation is urgently needed [3,6].

This new information about the diversity in its population genetics demonstrates the degree of success these plants have had in adapting to their altered environments over time. Therefore, it is of critical importance that this knowledge be applied in assessing their conservation value and the status of particular populations when developing management plans [32,33]. Despite dramatic reductions in natural habitat, based on our results, the high genetic diversity is still maintained within populations of *D. membranaceus* in Yunnan, China. However, the genetic differentiation among the populations and the limited reproductive capability of *D. membranaceus* in the wild indicate that it is still necessary for us to protect all of the existing natural populations and their habitat in Yunnan, China. At the same time, germplasm collection and conservation in the Lancang-Mekong River Valley, the distributional center of this species, should be placed at the first and foremost position.

## 3. Experimental Section

### 3.1. Plant Materials

**Figure 5:**
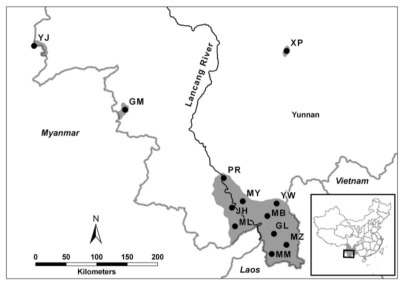
Distribution of extant natural *Dendrocalamus membranaceus* forests (shaded) and locations of 12 populations sampled in Yunnan Province, China.

A total of 12 natural populations were sampled from almost all of the main growing regions of *Dendrocalamus membranaceus* in Yunnan Province, China (Table 1, Figure 5). Leaves were collected from 240 clumps (20 per population) that were at least 100 m apart at each site. Using the method of McClure [24], we regarded each clump as a potential genet and the culms within as ramets of a clone, and we assumed that an assortment of genetic material existed within each population. All of the samples were dried with silica gel and stored at 4 °C prior to DNA extraction. Vouchers for each population were deposited at the Herbarium of the Southwest Forestry University (SWFC), Kunming, Yunnan, China.

### 3.2. Total DNA Extraction

Genomic DNA was extracted according to the CTAB protocol [34]. The quality of DNA was determined using 1.0% agarose gels. The purified genomic DNA was quantified using a BioRad Smartspec3000 UV-Vis spectrophotometer.

### 3.3. ISSR PCR Amplification

Genomic DNA was PCR-amplified using ISSR primers obtained from the Biotechnology Laboratory, University of British Columbia (Vancouver, Canada). Amplifications were performed using an ABI 2720 Thermal Cycler (Applied Biosystems, USA). The 20 μL mixture contained 10 ng of template DNA, 2.0 μL of 10 × PCR buffer, 1.8 mM MgCl_2_, 0.1 mM dNTPs (TAKARA, Dalian, China), 2% formamide, 100 nM of each primer, 1.5 units of Taq polymerase (Fermentas), and double-distilled water. The cycling conditions included an initial denaturation at 95 °C for 3 min; then 40 cycles of 95 °C for 30 s, 50 °C for 30 s, and 72 °C for 2 min; and a final extension at 72 °C for 10 min. The PCR products were electrophoretically separated using 2.0% agarose gels buffered with 0.5× TBE. A 100-bp DNA ladder (Fermentas) was used as a size marker. The PCR reactions were repeated twice to ensure reproducibility. After staining with ethidium bromide (1 μg·mL^−1^), the DNA fragments were identified by software Lab Works Software 3.0 [35] for gel documentation.

### 3.4. Data Analysis

Only distinct, reproducible, well-resolved fragments were scored as present (1) or absent (0) for each marker, and were displayed as part of a binary matrix. The data matrices were analyzed using POPGENE version 1.31 [36]. The following genetic parameters were determined: The percentage of polymorphic bands (PPB), expected heterozygosity (*H*_E_), coefficient of gene differentiation (*G*_ST_), genetic distance (*D*), and Shannon diversity index (*S*). An analysis of Molecular Variance (AMOVA program Version 1.55) [37] was performed to describe the genetic structure and variation among the populations and among the individuals. The number of private bands was calculated using GenAlEx 6.1 [38].

To visualize the genetic relationships among the populations, we constructed a dendrogram based on Nei’s [39] genetic distance *D*, implementing an unweighted pair-group method of cluster analysis that used arithmetic averages (UPGMA) and the software program NTSYS-PC V2.1 [40]. To test for putative correlations between the genetic distances and geographic distances among the populations, we performed Mantel tests using *TFPGA* software [41], computing 1000 permutations.

## 4. Conclusions

In summary, high genetic diversity and lower genetic differentiation were detected among the *Dendrocalamus membranaceus* populations in Yunnan. A large proportion of the genetic variation (78.95%) resides among individuals within the populations, whereas only 21.05% exist among the populations. No significant correlation was found between the genetic and geographic distances among the populations. Considering that *D. membranaceus* is a monocarpic and has prominently low sexual reproduction, we propose to implement *in situ* conservation measures for all of the populations and collect sufficient samples for *ex situ* conservation. Our results are also of important significance for the resource conservation of *D. membranaceus* in its main natural habitat, the Lancang-Mekong River Valley.

## Figures and Tables

**Table 1:** Populations of *Dendrocalamus membranaceus* examined in the ISSR analysis.

Population Code	Location in Yunnan Province	Elevation (m)	Sample Size (Number of Clumps)	Geographic Coordinates
YJ	Tongbiguan, Yingjiang	310	20	24°26′32″N, 97°32′51″E
XP	Gasa, Xinping	660	20	24°21′03″N, 101°37′50″E
GM	Mengding, Gengma	532	20	23°30′25″N, 99°01′13″E
PR	Simaogang, Pu’er	636	20	22°29′24″N, 100°35′32″E
MY	Mengyang, Jinghong	905	20	22°08′38″N, 100°53′18″E
YW	Yiwu, Mengla	844	20	22°06′13″N, 101°25′36″E
JH	Jinghong, Jinghong	638	20	22°03′04″N, 100°43′03″E
MB	Manbian, Mengla,	550	20	21°55′17″N, 101°16′39″E
ML	Menglong, Jinghong	723	20	21°46′18″N, 100°45′41″E
GL	Guanlei, Mengla	785	20	21°39′29″N, 101°22′41″E
MZ	Manzhuang, Mengla	625	20	21°29′32″N, 101°34′22″E
MM	Mengman, Mengla	788	20	21°21′41″N, 101°20′19″E

**Table 2:** Sequences and numbers of bands for 10 primers.

Primer	Sequence 5′→3′	No. of Amplified Bands	% Reproducibility of Bands	No. of Polymorphic Bands	% Polymorphic Bands (PPB)
UBC 807	(AG)_8_T	19	95	18	95
UBC 810	(GA)_8_T	17	92	17	100
UBC 841	(GA)_8_YC	18	94	18	100
UBC 853	(TC)_8_RT	13	94	13	100
UBC 855	(AC)_8_YT	15	96	14	93
UBC 857	(AC)_8_YG	12	91	12	100
UBC 859	(TG)_8_RC	16	93	16	100
UBC 864	(ATG)_6_	15	90	15	100
UBC 873	(GACA)_4_	18	92	18	100
UBC 878	(GGAT)_4_	12	88	12	100
Total		155	93	153	99

**Table 3:** Genetic variability within populations of *Dendrocalamus membranaceus.*

Population Code a	% Polymorphic Bands (PPB)	Expected Heterozygosity *H*_E_ (SD)	Shannon’s Diversity Index *S* (SD)	Number of Private Bands
YJ	49	0.165 (0.194)	0.248 (0.281)	2
XP	39	0.150 (0.205)	0.219 (0.293)	0
GM	55	0.186 (0.202)	0.279 (0.287)	4
PR	50	0.167 (0.194)	0.252 (0.280)	2
MY	49	0.173 (0.199)	0.259 (0.287)	3
YW	51	0.179 (0.201)	0.267 (0.288)	0
JH	47	0.156 (0.192)	0.236 (0.277)	0
MB	52	0.176 (0.204)	0.263 (0.290)	7
ML	51	0.162 (0.191)	0.264 (0.275)	3
GL	39	0.146 (0.199)	0.216 (0.287)	2
MZ	50	0.162 (0.192)	0.246 (0.277)	1
MM	45	0.147 (0.187)	0.220 (0.272)	0
Mean (SD)	48	0.164 (0.013)	0.249 (0.021)	

a Population codes are explained with Table 1.

**Table 4:** Analysis of molecular variance (AMOVA) for inter-simple sequence repeat (ISSR) variation for *Dendrocalamus membranaceus* populations.

Source of Variation	Degrees of Freedom	Sum of Squares	Mean Squares	Variance Components	% Total Variance	*p*-Value
Among populations	11	114.58	10.42	0.44	21.05	<0.001
Within populations	228	375.0	1.64	1.64	78.95	<0.001
Total	239	489.58		2.08		

**Table 5:** Geographic distance (km) (above diagonal) and genetic distance (below diagonal) between populations of *Dendrocalamus membranaceus*.

	YJ a	XP	GM	PR	MY	YW	JH	MB	ML	GL	MZ	MM
YJ	-	411	203	380	408	470	404	495	447	512	529	523
XP	0.094	-	261	238	252	239	261	296	320	314	325	334
GM	0.060	0.103	-	178	207	268	203	294	252	311	328	324
PR	0.050	0.109	0.071	-	30	96	30	117	92	135	151	149
MY	0.062	0.099	0.067	0.067	-	74	12	87	69	105	121	119
YW	0.064	0.116	0.074	0.073	0.075	-	84	57	106	74	86	94
JH	0.052	0.091	0.063	0.049	0.040	0.056	-	92	63	108	125	121
MB	0.084	0.150	0.101	0.048	0.091	0.087	0.072	-	74	18	34	38
ML	0.048	0.113	0.072	0.041	0.063	0.058	0.029	0.062	-	80	94	82
GL	0.095	0.105	0.104	0.083	0.091	0.094	0.060	0.101	0.069	-	17	20
MZ	0.053	0.099	0.088	0.063	0.070	0.062	0.050	0.090	0.052	0.045	-	16
MM	0.072	0.066	0.086	0.069	0.072	0.094	0.049	0.102	0.064	0.086	0.074	-

a Population codes are explained with Table 1
